# New Insights Into the Role of Phenotypic Plasticity and EMT in Driving Cancer Progression

**DOI:** 10.3389/fmolb.2020.00071

**Published:** 2020-04-23

**Authors:** Sugandha Bhatia, Peiyu Wang, Alan Toh, Erik W. Thompson

**Affiliations:** ^1^Institute of Health and Biomedical Innovation and School of Biomedical Sciences, Queensland University of Technology, Brisbane, QLD, Australia; ^2^Translational Research Institute, Brisbane, QLD, Australia

**Keywords:** EMT, EMP, stem cell, CTCs, hybrid EMT states, metastasis, metabolism, tumor cell heterogeneity

## Abstract

Tumor cells demonstrate substantial plasticity in their genotypic and phenotypic characteristics. Epithelial-mesenchymal plasticity (EMP) can be characterized into dynamic intermediate states and can be orchestrated by many factors, either intercellularly via epigenetic reprograming, or extracellularly via growth factors, inflammation and/or hypoxia generated by the tumor stromal microenvironment. EMP has the capability to alter phenotype and produce heterogeneity, and thus by changing the whole cancer landscape can attenuate oncogenic signaling networks, invoke anti-apoptotic features, defend against chemotherapeutics and reprogram angiogenic and immune recognition functions. We discuss here the role of phenotypic plasticity in tumor initiation, progression and metastasis and provide an update of the modalities utilized for the molecular characterization of the EMT states and attributes of cellular behavior, including cellular metabolism, in the context of EMP. We also summarize recent findings in dynamic EMP studies that provide new insights into the phenotypic plasticity of EMP flux in cancer and propose therapeutic strategies to impede the metastatic outgrowth of phenotypically heterogeneous tumors.

## Introduction (EMT-MET)

Epithelial–mesenchymal transition (EMT), in which epithelial cells undergo dynamic cellular transition from a sessile epithelial state to a motile mesenchymal state allowing the formation of new tissues, is considered one of the pivotal processes during embryogenesis and organogenesis (Chaffer et al., 2007; Yang and Weinberg, 2008). The process of EMT (classified as three different subtypes) has been implicated in a broad range of normal and pathophysiological processes from development, wound healing and tissue regeneration (type I), to organ fibrosis (type 2), and cancer progression (type 3) (Kalluri and Weinberg, 2009). During cancer progression, it is postulated that epithelial-derived carcinoma cells undergo a reversible, trans-differentiation process with changes in cell–cell adhesion and polarity, cytoskeletal remodeling, migratory and invasive enhancement, and dissemination into secondary organs via local invasion, intravasation and transfer through the blood stream and lymphatics (Polyak and Weinberg, 2009). In addition to cellular migration during metastasis, EMT also influences resistance to anoikis and apoptosis, blocks senescence, enhances survival, facilitates genomic instability, causes cancer stem cell (CSC) activity, alters metabolism, and induces drug resistance and immune suppression (Przybylo and Radisky, 2007; Ansieau et al., 2008; Gal et al., 2008; Kumar et al., 2011; Huang et al., 2013; Dongre et al., 2017; Lee et al., 2018; Redfern et al., 2018).

After invasion and spread, cancer recurrence at the metastatic site is thought to require the reverse process, termed mesenchymal to epithelial transition (MET) (Chaffer et al., 2007; Hugo et al., 2007; Brabletz, 2012). The reversal of EMT, referred to as MET, has received less attention than EMT in the establishment of metastasis. Microenvironmental cues are considered a major deterministic factor for the reversion of the migratory mesenchymal neoplastic cells and the subsequent development of macrometastases. However, the re-expression of E-cadherin, inhibition of SNAIL, and β-catenin sequestration have provided evidence of MET in liver metastasis from MDA-MB-231 (Chao et al., 2010; Brabletz, 2012), as has the anti-metastatic effects of sustained pro-mesenchymal signals (Ocana et al., 2012; Tsai et al., 2012). The concept of MET in metastasis is refuted in some of the cancer recurrence studies as no definitive proof of a MET requirement was obtained in the MMTV-PyMT genetically engineered mouse model (GEMMs) of metastatic breast cancer or in the KPC GEMM for metastatic pancreatic cancer (Fischer et al., 2015; Zheng et al., 2015). Nevertheless, recent data on EMP phenomena during metastatic cancer colonization is emerging (Chao et al., 2010; Rhim et al., 2012; Nieto, 2013; Beerling et al., 2016; Pastushenko et al., 2018) and could be of particular interest in breast and pancreatic carcinomas where EMT is considered an early event in tumorigenesis (Hüsemann et al., 2008; Rhim et al., 2012). Moreover, other studies have reported at least partial involvement of EMP in the breast model (Ye et al., 2015) and Zeb1 has been shown to contribute to metastasis in the pancreatic model (Krebs et al., 2017).

Considerably less information is available on the key intrinsic factors that drive MET *in vivo* and *in vitro*, while the drivers and transcriptional mediators of EMT are quite comprehensively documented (Stemmler et al., 2019). Bone morphogenetic protein 7 (BMP7) is reported to trigger MET in renal fibroblasts during kidney development (Zeisberg et al., 2005), and also in breast cancer cells, reducing their capability to form bone metastases (Buijs et al., 2007). Protein Kinase A was recently identified as an inducer of MET in human mammary epithelial cells (Pattabiraman et al., 2016). The role of Notch4 in melanoma cells to induce MET and suppress malignancy in mice has also been reported (Bonyadi Rad et al., 2016). The course of epigenetic reprograming is also supporting EMT and MET acquisition (Tamura et al., 2000). Reversible epigenetic changes acquired during EMT underpin the emergence of self-renewal and chemo-refractory stem cell-like features, which can revert to the MET phenotype for establishing metastasis (Voulgari and Pintzas, 2009; Sharma et al., 2010). Here, we discuss the role and the regulatory mechanisms of EMP, with the focus on recent emerging concepts that highlights the bidirectional dynamics of this phenomenon and the hybrid intermediate states. We also provide a brief overview of various techniques/modalities employed to analyze EMP in cancer. Understanding the phenotypic plasticity will provide insights for various therapeutic strategies that can be implemented to prevent/restrict spread of cancer by metastasis.

## Significance of EMP and Hybrid EMT States

Epithelial–mesenchymal transition, however, is not a two-step event through which cancer cells lose epithelial markers and acquire mesenchymal traits between two rigid phenotypes. Rather, studies performed within the last decade increasingly show that cancer cells sequentially acquire mesenchymal traits, but don’t automatically dissipate all of their previously expressed epithelial features (Tam and Weinberg, 2013; Aiello and Kang, 2019). The term “epithelial–mesenchymal plasticity” (EMP) is more favored recently as compared to EMT-MET (Bhatia et al., 2017; Williams et al., 2019). The multiple signal transduction cascade for EMT-MET programing results in dynamic and intermediate transitional states wherein, the cancer cells can reside in all three EMP phenotypes (epithelial, mesenchymal and hybrid phenotype). EMP reflects the bidirectional flux often in a continuum across the full spectrum (Lee et al., 2006; Oltean et al., 2006). Thus, a full spectrum of EMP endows the formation of a new carcinomatous tumor at distant organ sites with similar histopathology as observed in primary tumor (Gunasinghe et al., 2012).

Hybrid epithelial-mesenchymal features of carcinoma cells have indeed been observed in various invasive carcinoma model systems (Lee et al., 2006; Klymkowsky and Savagner, 2009), in which individual cells co-express markers of both epithelial and mesenchymal lineages, and circulating tumor cells (CTCs) in particular have been shown to exhibit a spectrum of EMP states (Armstrong et al., 2011; Yu et al., 2014; Khoo et al., 2015; Bourcy et al., 2016); reviewed in McInnes et al. (2015); Hassan et al. (2020). The hybrid EMP state seen in carcinomas and CTCs, in which individual cells co-express markers of both epithelial and mesenchymal lineages, is predicted to have the highest tumourigenicity and metastatic potential (Lee et al., 2006; Klymkowsky and Savagner, 2009; Jolly et al., 2016; Kroger et al., 2019; Pastushenko and Blanpain, 2019). An emerging challenge is also to decipher correctly the contribution that intermediate states of the EMT spectrum make to tumor evolution for therapeutic interventions.

## Extrinsic and Intrinsic Mechanisms and Regulators Involved in Plasticity

**FIGURE 1:**
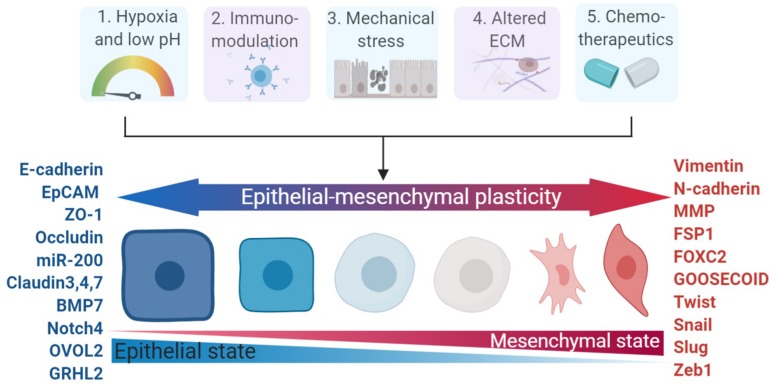
Major categories of EMP stimuli and markers involved in EMP. The dynamics of the epithelial – mesenchymal spectrum can be induced by five major stimulii (hypoxia, immuno-modulators, mechanical stress, altered ECM, and chemotherapeutics), which involve changes in various functional and morphological states and enlisted markers across the spectrum of epithelial–mesenchymal plasticity. ECM, extracellular matrix.

The crosstalk mediated by autocrine and/or paracrine factors secreted by cancer cells and tumor stroma has been widely proven to occur via extracellular mediators of EMT (Scheel et al., 2011). A host of extracellular mediators secreted by tumor stromal cells are already proven to elicit EMT induction. Examples of validated extracellular mediators as EMT inducers include TGF-β (Buonato et al., 2015), EGF (Hugo et al., 2009), FGF (Kurimoto et al., 2016), PDGF (Devarajan et al., 2012), HGF (Suarez-Causado et al., 2015), IGF (Wang et al., 2016), Interleukin-6 (IL-6) (Miao et al., 2014), WNT (Ochoa-Hernandez et al., 2012), Hedgehog (Yoo et al., 2011), and Notch (Yuan et al., 2014). Other inducers of EMT include collagen types I and III, matrix metalloproteinases-2 (MMP-2), MMP-3, MMP-9, and MMP14/MT1-MMP (Thiery et al., 2009). YAP and TAZ are also emerging as key modulators in inducing plasticity and skin cancer initiation (Moroishi et al., 2015; Debaugnies et al., 2018). EMT of tumor cells can also be induced by various stimuli from the tumor microenvironment (Marcucci et al., 2014); Fabrizio Marcucci and his colleagues proposed five major classes of these stimuli in 2016 (Marcucci et al., 2016): hypoxia and low pH, innate and adaptive immune responses, mechanical stress, altered ECM and treatment with chemotherapeutics (Figure 1).

Interestingly, hypoxic features in the tumor microenvironment can stimulate EMT as a downstream consequence of upregulated hypoxia-inducible factor 1α (HIF1α) (Wong et al., 2012). Apart from tumor microenvironment stimuli for EMT induction, stimulus-independent activation of signaling pathways, caused by mutations or epigenetic modifications leading to overexpression of certain pathway components, can also trigger EMT (Wallin et al., 2012; Serrano-Gomez et al., 2016). Gain-of-function mutations in P53 has been reported to induce EMT via modulation of miR-130b-Zeb1 axis (Dong P. et al., 2013).

Epigenetic modifications can also cause a shift of epithelial to mesenchymal state; for example, aberrant DNA CpG island methylation correlated with the repression of the miR-200 cluster, which promotes EMT and contributes to tumor progression (Vrba et al., 2010). LSD1-dependent genome-scale epigenetic reprograming was also observed during EMT (McDonald et al., 2011; Tang et al., 2013; Boulding et al., 2019). Various other chromatin regulators (e.g., DNMT1, KDM6B, PHF8, EZH2, and HDAC) are also reported to regulate EMT, genomic stability and metastasis (Suvà et al., 2013; Lu and Kang, 2019). Apart from epigenetics and mutations, EMT can also be modulated at transcriptional, post-transcriptional, translational and post-translational levels. The intrinsic gene network regulators, via alternate splice isoforms of ESRP1/2, microRNAs and long non-coding RNAs, also acts as other distinctive mechanisms to induce EMT (Aiello et al., 2018; Aiello and Kang, 2019). It has been postulated that during chemotherapy regimens, undifferentiated cancer cells also commence EMT, causing therapy resistance, CSC-like behavior, and a high propensity for metastasize. Tumor relapse after drug treatment cessation is due to persistence of disseminated CSC with mesenchymal features (Witta et al., 2006). Redfern et al. (2018) have also recently shown shorter overall survival times in patients treated with EMT-inducing agents compared to agents known to inhibit EMT.

The expression changes of various key molecular markers during EMT, are represented in Figure 1 (Christiansen and Rajasekaran, 2006; Pastushenko and Blanpain, 2019). The transition of epithelial cells to a more mesenchymal state is also characterized by reduced intracellular adhesion through the downregulation of E-cadherin (CDH1) and EpCAM, and gain of mesenchymal markers such as N-cadherin (CDH2), vimentin and FSP1/S100A4 (Francart et al., 2018). Repressors of E-cadherin can be divided into groups that modulate either directly or indirectly effects on gene transcription by binding to promoter sites. ZEBs, SNAIL1 and KLF8 repress expression by binding the E-cadherin promotor, thereby inactivating transcription, while E2.2, FOXC2, GOOSECOID, and TWIST repress E-cadherin transcription as indirect repressors (Peinado et al., 2004, 2007; Xu et al., 2019). These factors also share an elaborate interactome, in that SNAIL1 upregulates SNAIL2 and TWIST (Thuault et al., 2008; Smit et al., 2009), SNAIL1 and TWIST then induce ZEB1 and SNAIL2 (Casas et al., 2011; Dave et al., 2011), and SNAIL2 induces ZEB2 (Thuault et al., 2008). Although commonly serving as repressors of E-cadherin, these broader mechanisms also selectively modulate other programs involved in cell division, cell survival, and cell attachment, thereby resulting in a motile, invasive and resistant cell phenotype (Barrallo-Gimeno and Nieto, 2005).

## Role of EMT in Tumor Initiation, Progression and Metastasis

Although much less studied than later tumor stages, a number of studies have made a connection between the linkage of EMT to stemness and tumor-initiating capacity (Mani et al., 2008; Morel et al., 2008). In some carcinoma cells, overexpression of EMT transcription factors (EMT-TFs) has been observed to drive and enhance tumorigenicity (Wellner et al., 2009), and in particular, EMT has been shown to cause avoidance of oncogene-induced senescence (Ansieau et al., 2008). In a mouse skin SCC model, low levels of TWIST was explicitly responsible for the tumor initiation process, whereas higher levels of TWIST induced EMT and tumor progression (Beck et al., 2015). In recent lineage tracing studies along with transcriptional and epigenomic profiling, Latil et al found disparities in the tumors generated from interfollicular epidermis (IFE) and hair follicle (HF) stem cells (Lgr5CreER). While IFE tumors showed a well-differentiated phenotype, tumors generated from HF stem cells displayed an EMT spectrum and increased metastatic potential (Latil et al., 2017).

The profound role of EMP in tumor progression and metastasis *in vivo* has remained a topic with various controversies (Brabletz et al., 2018; Williams et al., 2019). The number of mesenchymal cells observed in primary cancers in many xenograft studies had been observed to be less than 10%. Although the specific dissemination process of these cells is not yet well documented (Bhatia et al., 2019; Lourenco et al., 2020), enrichment of EMT in circulating tumor cells has supported a role for EMT in the initial steps of metastasis. Various studies have highlighted the role of key EMT TFs, such as Slug and Zeb1, in promoting metastasis of breast and colorectal cancer to liver and lung, respectively (Spaderna et al., 2008; Guo et al., 2012). Downregulation of TWIST expression in highly metastatic mammary carcinoma cells was found to inhibit their metastatic seeding ability in the lung (Yang et al., 2004). However, these studies are nuanced by observations that enforced overexpression or downregulation of EMT-TFs doesn’t recapitulate the dynamic spectrum of transitional and/or partial EMT states discovered *in vivo* (Pastushenko et al., 2018). Similarly, the studies from the genetic abrogation of Twist or Snail in mouse models of pancreatic adenocarcinoma and from EMT lineage tracing using Fsp1 and β-actin promoter in breast cancer mouse model have questioned the indispensability of full mesenchymal transition in the metastasis process (Fischer et al., 2015; Zheng et al., 2015). The conclusions of these studies have been subsequently refuted by other studies where genetic depletion of Zeb1 in the same pancreatic model resulted in strong suppression of metastasis. Therefore, caution is required while interpreting such results as the context of EMT and other compensatory mechanisms may significantly influence their role in promoting metastasis (Aiello et al., 2017; Ye et al., 2017). With the advent of cell fate mapping studies using intra-vital imaging, plasticity was revealed in mouse breast tumor cells from primary site to its re-epithelisation upon metastasis (Beerling et al., 2016). Several other studies have also reported the direct evidence of EMP under physiological conditions (Rhim et al., 2012; Chaffer et al., 2013; Ye et al., 2015). Multiple tumor subpopulations screened from mammary and skin tumors suggested that tumor cells with hybrid phenotypes were more efficient in dissemination and metastasis (Pastushenko et al., 2018; Thompson and Nagaraj, 2018; Pastushenko and Blanpain, 2019; Rios et al., 2019). Similar, other relevant studies are also emerging to suggest that cancer cells mostly transition between epithelial/mesenchymal and hybrid intermediate states, but rarely undergo complete EMT during metastasis (Kroger et al., 2019).

## EMP Analysis of Circulating Tumor Cells (CTCs)

Generation of CTCs is regarded as a consequential effect of the multi-step processes that constitute the metastasic cascade (Lambert et al., 2017), and have become a particularly rich source of evidence and information regarding the role of EMP in cancer progression. Understanding the biology and characteristics of CTCs can provide important insights into the molecular and cellular requirements of cancer cells during metastatic spread. Observations of enriched levels of mesenchymal genes (e.g., N-cadherin, vimentin and Twist) and reduced expression of epithelial genes (e.g., E-cadherin, EpCAM and CK8/18/19) has been reported in the CTCs relative to cells in the tumors of origin in the breast cancer patients (Yu et al., 2013; Wang et al., 2018). Although many CTCs exhibit a mesenchymally enriched phenotype, some researchers have revealed that a small population of CTCs co-expressed both epithelial and mesenchymal (E/M) hybrid phenotype traits, which likely promoted cell migration, cell invasion and cell survival capabilities (Lecharpentier et al., 2011; Milano et al., 2018). Hence hybrid CTCs may be more metastatic than mesenchymal CTCs.

High numbers of CTCs in blood is significantly associated with poor prognosis in several carcinoma types, such as prostate cancer (Wang et al., 2011), breast cancer (Bulfoni et al., 2016), pancreatic cancer (Han et al., 2014), lung cancer (Naito et al., 2012), and increasingly these have taken account of CTC phenotypes (Tachtsidis et al., 2016). Pan et al. (2019) conducted a correlation study between CTC phenotypes and clinicopathological features of early cervical cancer, finding lower CTC counts in stage I patients than stage II patients with pelvic lymph node metastasis, but also that mesenchymal CTCs expressing vimentin and TWIST were more commonly found in the latter. Consistently, Markiewicz et al. (2014) selectively found of *VIM*, *SNAI1*, and *UPAR* expression in mesenchymal CTCs derived from breast cancer patient with lymph nodes metastases. Due to the low number of CTCs in blood, the greatest challenge in studying CTCs is the detection and isolation of these cells from patients’ blood (Kowalik et al., 2017). Molecular profiling of EMT markers in CTCs has been used to establish tools to isolate and classify CTCs. RNA *in situ* hybridization (RNA-ISH) is a detection method that employs specific probes targeting different epithelial and mesenchymal genes to detect multiple transcripts simultaneously (Lopez-Munoz and Mendez-Montes, 2013). An enhanced RNA-ISH-based detection system, CTCscope, was innovated to detect eight epithelial markers and three EMT markers (Payne et al., 2012), and has been employed successfully in the landmark breast cancer CTC study (Yu et al., 2013; Wang et al., 2018). The FDA-approved CELLSEARCH^®^ system (Menarini-Silicon Biosystems, Inc.), which immunocaptures EpCAM-expressing CTCs for patient prognosis (Riethdorf et al., 2007), is intrinsically biased toward predominantly epithelial CTCs. However, recent CTC studies have employed microfluidic devices to capture and isolate CTCs according to their size and deformability, which allows for better coverage of different phenotypic states (Lemaire et al., 2018; Ribeiro-Samy et al., 2019).

Although the devices used to isolate CTCs have improved the quality and quantity assessment of CTCs, there are still limitations when studying CTCs. Over the past few years, use of the revolutionary single- cell RNA sequencing (scRNA-seq) has emerged to assess genome-wide expression profiles of isolated CTC populations and CTC clusters. Aceto et al. (2014) conducted scRNA-seq on endogenous CTCs generated using tumor xenografts of LM2 variant of MDA-MB-231 human breast cancer cells, showing that CTC clusters are oligoclonal and highly metastatic compared to single CTCs. It was found that the cell junction protein plakoglobin (JUP) mediates cell cluster formation, enhancing the metastatic potential of CTCs. Ting et al. (2014) performed scRNA-seq analysis on CTCs in a mouse pancreatic cancer model, and revealed a universal loss of the epithelial markers E-cadherin (*Cdh1*) and Mucin-1 (*Muc1)* across all CTCs compared with the primary xenograft tumors. Hugo et al. (2017) showed that both *in vitro* and *in vivo* knockdown of *Cdh1* in MDA- MB-468 breast cancer cells reduced proliferation, and this was also reported by Padmanaban et al. (2019), who further indicated that the loss of *Cdh1* increased invasion capacity while reducing cell survival, CTC number and metastasis spread in the breast cancer.

The interconnection between CTC, EMT and CSC has been actively studied and reported to harbor important mechanisms underlying tumourigenicity (Agnoletto et al., 2019). EMT generates stem-like cells (Mani et al., 2008) and tumor cells that features both EMT and stem-like characters are better equipped to induce metastasis (May et al., 2011; Barriere et al., 2014), while some CTCs have dynamic cellular plasticity expressing EMT traits and stemnicity (Alonso-Alconada et al., 2014). A minor fraction of EMT hybrid phenotype CTCs have been shown to exhibit stem-like features, and these cells have been shown to promote collective migration Kaigorodova et al. (2017); Quan et al. (2020), as well as enhanced survivability and chemoresistant (Papadaki et al., 2019). Papadaki et al. (2019) modeled four CTC subpopulations based on the co-expression of three different markers; cytokeratin (epithelial marker), ALDH1 (stemness marker) and TWIST1 (partial EMT marker), and revealed that CTCs co-expressing cytokeratin, high levels of ALDH1, and nuclear TWIST1 (CSC^+^/partial-EMT^+^) were enriched after the first-line chemotherapy, implying that they were the most chemoresistant subpopulation, and had a favored prognostic value in patients with metastatic breast cancer. Another study has showed that EpCAM^high^ CTCs were significantly associated with poor prognosis compared to EpCAM^low^ CTCs in patients with breast and prostate cancer (de Wit et al., 2018), however the level of mesenchymal co-expression was not measured. Ting et al. (2014) showed that the stem cell markers *Aldh1a1* and *Aldh1a2* were enriched in pancreatic CTCs, and they also demonstrated that *Igfbp5* (a transport protein of epithelial stroma) and *SPARC* (a collagen-binding glycoprotein related to ECM reorganization) were highly expressed in the CTCs. Although they stated that there was no intrinsic correlation between EMP state and stemness in their CTCs, other reports have shown expression of these genes were associated with Cdh1 reduction (Bradshaw, 2009; Sureshbabu et al., 2012). There still remains a lack of evidence to fully elucidate the mechanistic relationship between CTCs, EMT and CSCs through the association of their existing markers with functional features, although it seems clear that they represent only a small fraction of CTCs.

## Understanding Dynamics of EMT

In the last two decades, many new concepts and findings have flourished around the dynamics of EMP. The dynamics of the stochastic state transitions, which allows cancer cells to switch between phenotypic states, is not yet explicitly described. However, novel concepts of dynamic equilibrium, asymmetrical dynamics of EMT-MET conversions, bet hedging, and hysteresis/cellular memory of cancer cells have heralded a deeper understanding of the phenotypic heterogeneity that cancer cells endow/possess (Jolly and Celià-Terrassa, 2019). This intrinsic mechanism of bi-directional transitions between epithelial (differentiated) and mesenchymal (stem-like) states is reported in different kinds of cancer (Polyak and Weinberg, 2009; Chaffer et al., 2011; Gupta et al., 2011; Yang et al., 2012; Ruscetti et al., 2016; Bhatia et al., 2019). Sequencing of breast cancer stem cell populations also indicates a dynamic conversion between differentiation states *in vivo* (Klevebring et al., 2014). A phenotypically stable equilibrium was observed in breast cancer cell lines, differentially segregated across cell state proportions (Gupta et al., 2011; Bhatia et al., 2019). DNA barcoding and subsequently high-throughput sequencing of breast cancer cell clones had also been employed to quantify the extent of intrinsic phenotypic plasticity exhibiting epithelial or mesenchymal phenotypes (Mathis et al., 2017; Rios et al., 2019). Various mechanism-based mathematical modeling and data-based statistical modeling approaches have been developed in an attempt to uncover the presence of these metastable states (Lu et al., 2013; Jolly et al., 2016; Jolly and Celià-Terrassa, 2019).

The presence of “multiple attractor states” based on Waddington landscape and intrinsic cellular variability also contributes to phenotypic plasticity (Huang et al., 2009; Ferrell, 2012; Li et al., 2016). The studies pertaining to EMT and MET reversion have also explained explicitly that the dynamics achieved for its reversion back may not follow the same path. For example, studies with a Snail-inducible expression system in prostate cancer cells has identified metabolic plasticity and asymmetrical dynamics during their EMT-MET cycle (Stylianou et al., 2019). Other studies, where re-expression of significant epithelial markers such as E-cadherin, OVOL2 and GRHL2 after their knockout may not obtain the same spectrum of reversion also suggests asymmetrical dynamics (Qi et al., 2018; Chung et al., 2019; Jolly et al., 2019). The concept of bet hedging had been observed in bacterial persistence under different environmental stimulations by generating mutation-independent phenotypic heterogeneity (Veening et al., 2008). This pre-existing phenotypic heterogeneity is thought to be exploited by cancer cells in generating drug-persistence cells via non-genetic mechanism, which might lead to anti-drug resilience in clinical scenarios (Jolly et al., 2018). The property of hysteresis and “cellular memory” allows cells from the same clonal population to respond differently to the same strength and duration of a signal. The differential response again can be attributed to the cellular placement across different “attractor states” or the possibility of history of input stimuli (Chang et al., 2006; Jolly and Celià-Terrassa, 2019). The possibility of EMT occurring via non-linear hysteretic mode had been recently observed to result in different dynamics and increased metastasis in a breast cancer model (Celià-Terrassa et al., 2018). Thus, these dynamics impart a further layer of intricacies in understanding the causes and reasons of non-genetic heterogeneity in cancer in regard to phenotypic plasticity. An integrative understanding of the approaches to block this phenotypic plasticity and EMP dynamics could further aid in combating cancer resistance.

## Implications of Metabolic Plasticity and EMP

During the processes of EMP, there are numerous adaptations, not only in cell morphology and epigenetic changes, but also in metabolism (Cha et al., 2015). Among them, glucose and lipid metabolism alterations are crucial for the EMT induction (Kondaveeti et al., 2015; Sánchez-Martínez et al., 2015; Morandi et al., 2017; Kang et al., 2019). In terms of carbohydrate metabolism, it is well known that cancer cells prefer to reply on the glycolysis to generate ATP instead of oxidative phosphoruylation (OXPHOS), even under the well-oxygenated conditions, according to the Warburg effect (Warburg, 1956). However, apart from the Warburg effect, other glucose metabolic pathway adaptations have been observed during the last decade. When cancer cells undergo an EMP process, their metabolism will reprogram from aerobic glycolysis for proliferation to EMT-like metabolism to meet the increased energy needs. Both enhanced glucose and lipid uptake and increased glycolytic mediated biosynthesis and lipid synthesis are the characteristics of EMT-like metabolism. The correlation between metabolism and EMP is dynamic. EMP-associated genetic changes can stimulate metabolic adaptations, while the higher metabolic rate can support and facilitate the EMP process.

A number of studies illustrate the EMT-associated metabolic changes and their implications. According to the research of Dong et al., up-regulation of the EMT-driving transcription factor Snail-1 in basal-like breast cancer cells leads to the formation of a Snail-G9a-Dnmt1 complex to silence the expression of fructose-1,6-bisphosphatase (FBP1), which is an important enzyme of gluconeogenesis (Dong C. et al., 2013). The loss of the FBP1 caused an increase in glucose uptake for ATP production and glycolytic mediated biosynthesis, like the pentose phosphate pathway (PPP), serine and glycerol-3-phosphate. The reprogramed metabolism offers enough energy to fuel the invasion and metastasis processes.

For lipid metabolism, higher expression levels of lipid synthesis enzymes such as ATP-citrate lyase (ACLY), stearoyl-CoA desaturase (SCD), fatty acid synthase (FASN) and HMG-CoA reductase, have been detected in more aggressive tumor cells (Sánchez-Martínez et al., 2015). Jing et al. reported that overexpression of these proteins in association with mutated p53 in mostly mesenchymal cancer cells, along with aberrant expression of sterol regulatory element-binding proteins (SREBPs) (Hu et al., 2013). In normal tissue, wild type p53 can inhibit the expression of SREBP-1c, a transcription factor of FASN and ACLY (Horton et al., 2002), while the mutated p53 loses this capacity. Moreover, the mutated p53 can bind with SREBP-2 to enhance the cholesterol biosynthesis (Freed-Pastor et al., 2012). Thus, mutated p53 significantly upregulates both fatty acid (FA) and cholesterol levels in cancer cells, which generate more membrane lipid rafts to support cell motility during the EMT process. High levels of SREBP1 can also induce EMT, via recruiting a SNAIL1/HDAC1/2 complex to stop E-cadherin mRNA expression (Zhang et al., 2019). Chen et al., has proposed that drugs targeting SREBPs could suppress cancer cell metastasis (Chen et al., 2018).

Growth factors from the tumor microenvironment can also reprogram cancer cells from the Warburg-like metabolism to EMT-like metabolism. Activated PI3K/AKT/mTOR signaling due to growth factor stimulation can enhance the uptake of glucose and lipid, as well as the synthesis of FA and protein (Chen et al., 2018). The study of EMP relative metabolism changes can offer a promising target for cancer therapy.

## Current Modalities to Investigate Plasticity

Many techniques recently employed in the field of cancer cellular plasticity have corroborated not only the epithelial and mesenchymal phenotypic states, but also the spectrum of intermediate and hybrid E/M states (Pastushenko et al., 2018; Karacosta et al., 2019). The molecular approaches widely used in the cancer EMT field are broadly divided into two categories: *in vitro* based molecular and functional assays and *in vivo* based cancer models. The *in vitro* assays routinely performed in EMP studies involve various molecular and functional assays. Molecular assays, using FACS and immunocytochemistry staining with microscopy analysis, relies on various validated EMP markers that are used to delineate the phenotypic state of cells (Celià-Terrassa et al., 2018; Pastushenko et al., 2018; Risom et al., 2018; Bhatia et al., 2019). Microscopy based snap-shot and real time analysis in conjunction with quantitative assessment is an imperative technique. These optic techniques are widely employed to study the cellular localization of various molecular markers, such as E-cadherin presence at the cell junctions, and also the subtle dynamic changes of various markers in the absence or presence of various stimuli or inducers can be studied (Hirata et al., 2014; Labernadie et al., 2017; Liu et al., 2018). Microscopy approaches are also well integrated in various functional assays, such as *in vitro* wound closure, Transwell migration studies performed in the presence or absence of ECM, quantification of single cell migration and invasion studies in culture medium, spheroid assessment and co-culture assays with cancer associated fibroblasts or endothelial cells (Kramer et al., 2013; Tanner and Gottesman, 2015; Mitchell and O’Neill, 2016; Klymenko et al., 2017; Reynolds et al., 2017; Mason et al., 2019). Other *in vitro* assessment also include “soft agar assay” for anchorage independent growth studies, “ECM degradation assays” to measure MMP and other protease activity, and “trans-epithelial resistance” assays to study monolayer integrity and permeability (Narai et al., 1997; Anderl et al., 2012; Borowicz et al., 2014). In studies relevant to single cell colonization, plasticity generated from single cell clonal culture is also examined for differences in migration, invasion and chemoresistance assays, which can be extrapolated to the metastatic cascade (Kramer et al., 2013; Harner-Foreman et al., 2017; Bhatia et al., 2019). While *in vitro* studies are important to study cellular behavior in context of phenotypic plasticity and tumoural non-genetic heterogeneity, these routinely performed assays have the drawback of not presenting the whole landscape of cancer and the real EMP spectrum, where cancer cells are infiltrated with stromal and immune microenvironment.

Researchers in the field of EMP have employed various animal models, including as *C. elegans*, *Drosophila Melanogaster*, chick embryos, zebrafish and mice to study the *in vivo* dynamics of phenotypic plasticity in developmental EMT and cancer EMP (Jimenez et al., 2016; Gómez-Cuadrado et al., 2017; Nieto, 2018; Stuelten et al., 2018; Campbell et al., 2019). Genetically engineered mouse models and patient-derived xenografts (PDXs) have been observed to recapitulate metastatic and organ homing properties similar to clinical specimens (Sikandar et al., 2017). Orthotopic implantation strategies, such as inoculation into the mammary fat pad, has also improved the recapitulation of the breast cancer in mice (Proia et al., 2011). In conjunction with intravital imaging and fluorophore chemistry, various Cre-Lox lineage tracing approaches have been employed in cell lines, and in injected mouse and zebrafish models, to delineate EMP status of the cells at primary and metastatic sites, and also of encaptured CTCs (Lourenco et al., 2020). These reporter tags are valuable in identification of CTCs and in scenarios of low numbers of cells seeding at secondary niches during metastasis (Zheng et al., 2015; Sikandar et al., 2017). The inducible system utilized for Twist1 induction or deletion at different stages of skin carcinogenesis allowed flexibility in spatio-temporal tuning (Tsai et al., 2012; Beck et al., 2015). The use of confetti mouse models and lineage tracing can also aid in the determination of intratumoural heterogeneity owing to clonal variations, and in fate mapping of the cancer evolution studies (Janiszewska and Polyak, 2018; Marx, 2018; Rios et al., 2019). Technological advances in the fields of single cell transcriptomic analysis (Patel et al., 2014; Ting et al., 2014; Horning et al., 2018; Kim et al., 2018; Puram et al., 2018; Cook and Vanderhyden, 2019), single-cell methylome profiling or ChIP sequencing (Rotem et al., 2015; Angermueller et al., 2016; Grosselin et al., 2019) and multiplex *in situ* imaging (Tsujikawa et al., 2017; Schulz et al., 2018) has allowed researchers to gain insightful information of cellular phenotypic status from clinical specimens. Microfluidic modalities are also gaining attention recently and are of great help not only in detection and capturing of label-free CTCs from patients, but also to gauge the effects of fluid pressures, cancer cell motility assessment associated with single cell or collective migration, and for co-culture studies (Sarioglu et al., 2015; Ma et al., 2018; Shang et al., 2019; Truong et al., 2019). Similarly, various mathematical approaches and modeling have been helpful in deciphering the significant genes and molecular networks associated with the spectrum of epithelial and mesenchymal states, as well as phenotypic plasticity (Jolly et al., 2017; Bocci et al., 2018; Jia et al., 2019; Yang et al., 2019). However, it is crucial to acknowledge that the modalities and analytical approaches utilized in the field of EMP present context-specific studies, such that inferences derived will not provide an overarching conclusion (Henkel et al., 2019). Inherent limitations of the employed assays should always be taken into consideration while extrapolating from the data.

## Therapeutic Strategies for Targeting EMP

**FIGURE 2:**
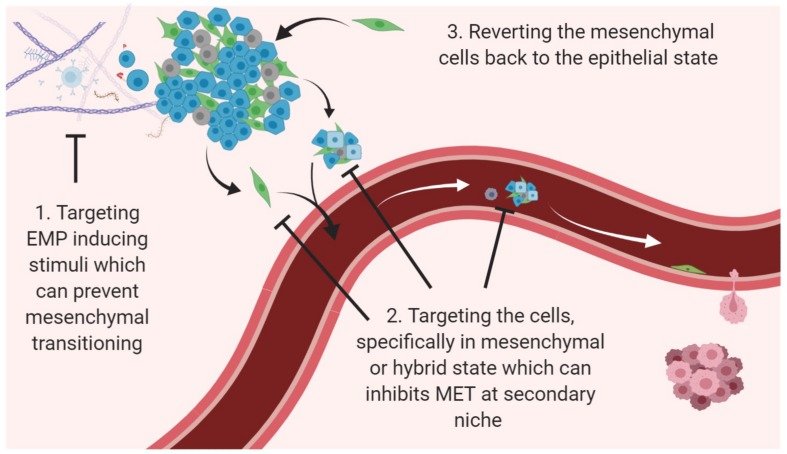
Potential avenues to target EMP. Three main strategies for targeting cancer progression and recurrence with relevance to EMP dynamics are to use agents/compounds (i) that can target the inducers to prevent EMT; (ii) that can selectively kill mesenchymal phenotype and cells present within multiple transition states; (iii) that can revert the cells via MET.

The presence of plasticity in tumor cells and resultant heterogeneity is one of the utmost challenges in targeting cancer on a whole (Bhatia et al., 2017; Redfern et al., 2018). EMT and/or CSC have been reported to confer drug resistance characteristics against a number of conventional therapeutics like taxol, vincristine, oxaliplatin, gemcitabine, cisplatin and 5-fluorouracil in human pancreatic cell lines, and against EGFR-targeted therapies erlotinib, cetuximab and gefitinib in lung cancer (Fuchs et al., 2008; Sabbah et al., 2008; Arumugam et al., 2009). Similarly, studies have also reported that an active EMT program in breast cancer cell lines makes them unresponsive to tamoxifen, paclitaxel, and adriamycin treatment (Kajita et al., 2004; Hiscox et al., 2006; Cheng et al., 2007; Li Q. Q. et al., 2009). Breast cancer cells with EMT-associated CSC features (CD44^high^, CD24^low^) have been reported to remain after neoadjuvant chemotherapy and HER2 pharmacological inhibition, suggesting that they encode resistance (Li et al., 2008; Blick et al., 2010). Many reports have also shown basal, mesenchymal-like neoplasms to be more resistant to neoadjuvant chemotherapy than epithelial, luminal-like tumors (Yauch et al., 2005; Carey et al., 2007; Liedtke et al., 2008), and reversal of the EMT phenotype in resistant cell lines has re-established drug sensitivity (Arumugam et al., 2009; Li Y. et al., 2009). Therefore, three main strategies as combinatorial therapies that are being widely acknowledged and/or proposed in the field of combating plasticity are (i) Targeting EMP inducing stimuli which can prevent mesenchymal transitioning, (ii) Targeting the cells, specifically in mesenchymal or hybrid state which can inhibit MET at secondary niche, and (iii) Reverting the mesenchymal cells back to the epithelial state (Bhatia et al., 2017; Williams et al., 2019; Figure 2).

In the first scenario to target EMP inducing stimuli, many different approaches have been utilized to inhibit different signaling pathways that contribute to the induction and maintenance of EMT, such as TGFβ/TGFβR, EGF/EGFR, FGF/FGFR, IGF/IGFR, IL-6/IL-6R, HGF/MET, PDGF/PDGFR, TNFα, Wnt and Notch signaling (Marcucci et al., 2016; Bhatia et al., 2017). Of all, TGFβ and EGF pathway inhibitors have been most extensively studied and investigated, as these have been found to be common inducers of EMT in different cancer types (Li et al., 2015). Table 1 details the current active clinical trials inhibiting these two EMT-inducing pathways in combination with chemotherapeutics.

**TABLE 1:** List of the current active clinical trials targeting EGF and TGF-β signaling pathways in combination with chemotherapeutics.

Target class	Functional class	Drug name	chemotherapeutics combination	Cancer type	Clinical status (first posted, recruitment status)	Intervention/treatment
TGF-β/TGF-β Receptor inhibitors	Tyrosine kinase inhibitor	LY-2152799 (Galunisertib)	Fluorouracil/Capecitabine + Tumor specific mesorectal excision	Locally Advanced Rectal Adenocarcinoma	NCT02688712 (2016, Recruiting)	Drug: LY2157299Drug: Capecitabine Drug: Fluorouracil Procedure: Tumor specific mesorectal excision
			Paclitaxel/Carboplatin	Carcinosarcoma of the Uterus or Ovary	NCT03206177 (2017, Recruiting)	Drug: Galunisertib Drug: Paclitaxel Drug: Carboplatin
			Sorafenib	Advanced Hepatocellular Carcinoma	NCT02178358 (2014, Active, not recruiting)	Drug: LY2157299 Drug: Sorafenib Drug: Placebo
	TGF-β receptor inhibitor	TEW-7197	Pomalidomide	Relapsed or Relapsed and Refractory Multiple Myeloma	NCT03143985 (2017, Recruiting)	Drug: TEW-7197 Drug: Pomalidomide
EGF/EGFR inhibitors	EGFR tyrosine kinase inhibitor	Gefitinib	Pemetrexed	Advanced Non-Small Cell Lung Cancer	NCT01982955 (2013, Active, not recruiting)	Drug: Tepotinib Drug: Gefitinib Drug: Pemetrexed Drug: Cisplatin Drug: Carboplatin
		Icotinib	Pemetrexed, Carboplatin	III B/IV Non-Small Cell Lung Cancer	NCT03151161 (2017, Not yet recruiting)	Drug: Icotinib, Pemetrexed, Carboplatin Drug: Icotinib
			Cisplatin or Carboplatin	metastatic non-squamous non-small cell lung cancer who did not progress after pemetrexed combined with platinum chemotherapy	NCT03992885 (2019, Recruiting)	Drug: Icotinib Drug: Cisplatin Drug: Carboplatin
		Apatinib	Pemetrexed, Gemcitabine, Docetaxel	NSCLC Patients Without T790M Mutation	NCT03758677 (2018, Not yet recruiting)	Drug: Apatinib Drug: Chemotherapy with platinum-based double drugs (Pemetrexed, Gemcitabine, Docetaxel)
			Pemetrexed Plus Carboplatin	Advanced Non-small Cell Lung Cancer	NCT03164694 (2018, Recruiting)	Drug: Apatinib + Pemetrexed + Carboplatin Drug: Pemetrexed + Carboplatin
		Osimertinib	Cisplatin or Carboplatin	Metastatic EGFR Mutant Lung Cancers	NCT03567642 (2018, Recruiting)	Drug: Osimertinib Drug: Platinum Drug: Etoposide
			Platinum-based Doublet-Chemotherapy	Locally Advanced or Metastatic Non-Small Cell Lung Cancer	NCT02151981 (2018, Active, not recruiting)	Drug: Chemotherapy Drug: Cross-over to Osimertinib
			Pemetrexed + Cisplatin or Pemetrexed + Carboplatin	Locally Advanced Non-Small Cell Lung Cancer	NCT04035486 (2019, Recruiting)	Drug: Osimertinib Drug: Osimertinib + Pemetrexed + Cisplatin Drug: Osimertinib + Pemetrexed + Carboplatin
	Monoclonal antibody	Panitumumab	Carboplatin and Paclitaxel	Invasive Triple Negative Breast Cancer	NCT02876107 (2016, recruiting)	Drug: Carboplatin Other: Laboratory Biomarker Analysis Drug: Paclitaxel Biological: Panitumumab
		HLX07	Gemcitabine + Cisplatin/Paclitaxel + Carboplatin/mFOLFOX6	Advanced Solid Tumors	NCT03577704 (2018, Recruiting)	Drug: HLX07 + Gemcitabine + Cisplatin Drug: HLX07 + Paclitaxel + Carboplatin Drug: HLX07 + mFOLFOX6

Secondly, for therapies specifically targeting mesenchymal cells, different novel strategies such as EMP-targeting vaccines against transcription factors such as TWIST1 and Brachyury; nutraceuticals; and the repurposing of drugs such as metformin, salinomycin and resveratrol, have been extensively discussed in our previous review (Bhatia et al., 2017). Table 2 details current clinical trials (2015 onward) with the focus on targeting EMP in cancer patients, as an update from our previous review (Bhatia et al., 2017). New combinatorial approaches combining EMT inhibitors alongside targeting immunotherapy blockade are also being developed, as EMT is reported to induce PDL1 expression in carcinoma cells (Chen et al., 2014; Noman et al., 2017), and an EMT signature was seen in tumors that responded to anti PD1/PD-L1- and CTLA4-associated treatments (Lou et al., 2016).

**TABLE 2:** Different categories of inhibitors that target stimuli and signaling pathways associated with EMT and are targeted in current clinical trials.

Target class	Functional class	Drug Name	Cancer type	Clinical status (first posted)
**Inhibitors of extracellular mediators and their corresponding receptors**				
TGF-β–TGF-β receptor inhibitors	TGF-β receptor inhibitor	TEW-7197	Urothelial Carcinoma Recurrent, Advanced Urothelial Carcinoma, Myelodysplastic Syndromes	NCT04064190(2019); NCT03074006(2017)
	TGFβ receptor ectodomain-IgG Fc fusion protein	AVID200	refractory advanced and metastatic malignancies, Myelofibrosis (Myeloproliferative Neoplasms Research Consortium [MPN-RC] 118)	NCT03834662(2019); NCT03895112(2019)
	a bifunctional fusion protein targeting PD-L1 and TGF-β	MSB0011359C (M7824)	Stage II-III HER2 Positive Breast Cancer, Locally Advanced or Metastatic Second Line (2L) Biliary Tract Cancer (Cholangiocarcinoma and Gallbladder Cancer), Solid Tumors, Recurrent Respiratory Papillomatosis, HPV Associated Malignancies	NCT03620201(2018); NCT03833661(2019); NCT02699515(2016); NCT02517398(2015); NCT03707587(2018); NCT03427411(2018)
	CAR-T cells that target GPC3 (GPC3-CART cell) and/or soluble TGFβ (GPC3/TGFβ-CART)	GPC3-T2-CAR-T	Hepatocellular Carcinoma, Squamous Cell Lung Cancer	NCT03198546(2017)
IL-6/IL-6R inhibitors	Monoclonal antibody	Siltuximab (CNTO-328, Tocilizumab)	Metastatic Pancreatic Cancer; multiple myeloma (MM) and systemic AL amyloidosis (AL)	NCT04191421(2019); NCT03315026(2017)
EGF/EGFR inhibitors	Tyrosine kinase inhibitor	Afatinib (BIBW2992)	Chordoma,	NCT03083678(2018)
		Dacomitinib (PF00299804)	EGFR Mutant Lung Cancer	NCT03755102(2018)
		Osimertinib	Stage I-IIIA EGFR-mutant Non-small Cell Lung Cancer, stage IIIB-IV or Recurrent Non-small Cell Lung Cancer	NCT03586453(2018); NCT03434418(2018); NCT03433469(2018); NCT03191149(2018)
		Brigatinib (AP26113)	Advanced Non-small Cell Lung Cancer (NSCLC), Anaplastic Large Cell Lymphoma, Advanced Malignant Neoplasm	NCT02737501(2016); NCT02706626(2017); NCT03719898(2018); NCT03868423(2019); NCT03707938(2018); NCT03596866(2019);
	inhibitor for (EGFR, HER2, and ErbB4)	Poziotinib (HM781-36B)	EGFR Exon 20 Mutant Advanced NSCLC, Breast Cancer, Stage IV Lung Adenocarcinoma with HER2 Mutation	NCT03066206(2017); NCT03066206(2017); NCT03744715(2018); NCT03318939(2017); NCT02979821(2016)
	Monoclonal antibody	Panitumumab	Anaplastic Lymphoma Kinase-Positive (ALK +), Advanced Non-Small-Cell Lung Cancer (NSCLC)	NCT03535740(2019)
		HLX07	Advanced Solid Cancers	NCT02648490(2016)
PDGF/PDGFR inhibitors	Tyrosine kinase inhibitor	Axitinib	Pheochromocytoma, Paraganglioma, Renal Cell Carcinoma, Hepatobiliary Neoplasm, Liver Neoplasm, Biliary Tract Neoplasms, Cervical Cancer, Non-Small Cell Lung Cancer, Urothelial Cancer	NCT03839498(2019); NCT03494816(2018); NCT04010071(2019); NCT03826589(2019); NCT03472560(2018); NCT03341845(2017)
FGF/FGFR inhibitors	Tyrosine kinase inhibitor	Lenvatinib	Advanced Biliary Tract Cancer, Thyroid Neoplasms, Advanced Gastric Cancer, Non-small Cell Lung Cancer, Solid Tumor, Thyroid Cancer	NCT04211168(2019); NCT03573960(2018); NCT03609359(2018); NCT03829332(2019); NCT03009292(2017); NCT03139747(2017)
		Nintedanib (BIBF1120)	Appendix Cancer, Lymphangioleiomyomatosis, Adenocarcinoma of the Lung	NCT03287947(2017); NCT03062943(2017); NCT04046614(2019)
		Pazopanib	Refractory Solid Tumors, Metastatic Sarcoma, Recurrent Sarcoma, Resectable Sarcoma, Advanced Renal Cell Carcinoma Metastatic Renal Cell Carcinoma	NCT02691767(2016); NCT04199026(2019); NCT03200717(2017)
		Ponatinib	Medullary Thyroid Cancer, Acute Myeloid Leukemia, Accelerated Phase Chronic Myeloid Leukemia, Blast Phase Chronic Myeloid Leukemia, GIST, Malignant, Chronic Myeloid Leukemia, Acute Lymphoblastic Leukemia, Philadelphia Chromosome-positive Acute Lymphoblastic Leukemia	NCT03838692(2019); NCT03934372(2019); NCT03171389(2017); NCT04233346(2020); NCT03709017(2018)
TNFα inhbitors	Monoclonal antibody	Infliximab	Advanced Melanoma	NCT03293784(2017)
Hedgehog/Smoothened inhibitors	Smoothened antagonists (small-molecule inhibitor)	Vismodegib	Stomach Neoplasms, Basal Cell Carcinoma, Metastatic Basal Cell Carcinoma, Locally Advanced Basal Cell Carcinoma, Advanced Solid Tumors	NCT03052478(2017); NCT03035188(2017); NCT03610022(2018); NCT03297606(2017)
		Sonidegib	Clinical Stage III Cutaneous Melanoma AJCC v8, Clinical Stage III Gastric Cancer AJCC v8, Basal Cell Carcinoma	NCT04007744(2019); NCT04066504(2019)
Notch/Notch ligand (Delta-like and Jagged) inhibitors	Small-molecule inhibitor	γ-secretase inhibitor: LY3039478	Advanced Solid Tumor	NCT02836600(2016)
		γ-secretase inhibitor: PF-03084014	Desmoid Tumor, Aggressive Fibromatosis, Desmoid-Type Fibromatosis, Recurrent Desmoid-Type Fibromatosis, Unresectable Desmoid-Type Fibromatosis	NCT03785964(2018); NCT04195399(2019)
		PAN-Notch inhibitor BMS-906024	recurrent or metastatic Adenoid Cystic Carcinoma	NCT03691207(2018)
WNT/Frizzled inhibitors	Wnt5a mimetic	Foxy-5	Colon Cancer	NCT03883802(2019)
	Peptidomimetics	CWP232291	Acute Myeloid Leukemia	NCT03055286(2017)
	Inhibits the recruiting of β-catenin with its co-activator CBP	PRI-724	Liver Cirrhosis	NCT03620474(2018)
**Inhibitors of intracellular signaling pathways**				
SRC inhibitors	Tyrosine Kinase inhibitor	Dasatinib (BMS-354825)	Relapsed AML, Waldenstrom Macroglobulinemia, Relapsed CML	NCT03560908(2018); NCT04115059(2019); NCT03573596(2018)
		Bosutinib (SKI-606)	Metastatic Breast Cancer, Chronic Myeloid Leukemia, Advanced Solid Tumors	NCT03854903(2019); NCT02810990(2016); NCT03297606(2017)
FAK inhibitors	Tyrosine Kinase inhibitor	Defactinib (VS-6063)	Malignant Pleural Mesothelioma, Advanced Solid Tumors	NCT04201145(2019); NCT02546531(2015)
PI3K/AKT/mTOR inhibitors	PI3K inhibitor	Idelalisib	Follicular Non-Hodgkin’s Lymphoma Refractory, Relapsed Diffuse Large B-cell Lymphoma, B-cell Lymphoma Recurrent, B-cell Chronic Lymphocytic Leukemia	NCT03568929(2018); NCT03576443(2018); NCT03757000(2018)
	AKT inhibitor	AZD5363	Advanced Solid Tumors, Advanced Breast Cancer	NCT03310541(2017); NCT03182634(2017)
		Temsirolimus	Non-muscle Invasive Bladder Cancer,	NCT02753309(2016)
	Tyrosine kinase inhibitor	CX-4945	Recurrent Medulloblastoma	NCT03904862(2019)
AURKA/SYK	Tyrosine kinase inhibitor	Midostaurin	Acute Myeloid Leukemia, AML/MDS	NCT03951961(2019); NCT04097470(2019)
AXL inhibitors	Tyrosine Kinase inhibitor	BGB324	Recurrent Glioblastoma Undergoing Surgery, Advanced NSCLC	NCT03965494(2019); NCT03184571(2017)
RAS/RAF/MAPK inhibitors	RAF inhibitor	Sorafenib	Recurrent or Metastatic Triple Negative Breast Cancer, Advanced Liver Cancer, Advanced Hepatic Carcinoma	NCT02624700(2015); NCT04163237(2019); NCT03164382(2017); NCT03211416(2017)
	MEK inhibitor	Trametinib	Advanced ALK-Positive NSCLC, Advanced/Metastatic Colorectal Cancer	NCT03087448(2017); NCT03714958(2018)
**Inhibitors of transcription factors that indirectly induce EMP**				
JAK and STAT3 inhibitors	Small molecule inhibitor	STAT3: BB1608 (Napabucasin)	Metastatic Colorectal Cancer, Metastatic Pancreatic Cancer	NCT03522649(2018); NCT03647839(2018); NCT03721744(2018)
**Compounds acting on epigenetic modulators**				
Histone deacetylase inhibitor		Vorinostat	Mutated Advanced Melanoma, Breast Cancer Metastatic	NCT02836548(2016); NCT03742245(2018)
		Romidepsin	Survey- Relapsed or Refractory Peripheral T-Cell Lymphoma	NCT03742921(2018); NCT03547700(2018)
		Mocetinostat	Advanced Lung Cancer, Unresectable Stage III or Stage IV Melanoma	NCT03220477(2017); NCT03565406(2018)
		Panobinostat	Multiple Myeloma	NCT02722941(2016); NCT04150289(2019);
Histone methyl transferases inhibitor	EZH2 inhibitor	E7438 (Tazemetostat, EPZ-6438)	Relapsed or Refractory B-cell Non-Hodgkin’s Lymphoma, Relapsed/Refractory Follicular Lymphoma	NCT03009344(2017); NCT03456726(2018); NCT04224493(2020)
	EZH1/2 inhibitor	DS-3201b	Relapsed or Refractory Adult T-cell Leukemia/Lymphoma, Acute Leukemia Myeloid Leukemia, Acute Lymphocytic, Recurrent Small Cell Lung Cancer	NCT04102150(2019); NCT03110354(2017); NCT03879798(2019)
**Inhibitors of stimuli from the tumor microenvironment**				
HIF-1α inhibitors	Small molecule inhibitor	PT2385	Von Hippel-Lindau Disease-Associated Clear Cell Renal Cell Carcinoma, Recurrent Glioblastoma	NCT03108066(2017); NCT03216499(2017)
		Digoxin	Breast Cancer, Circulating Tumor Cells (CTCs), Advanced Pancreatic Cancer, Advanced Solid Tumor	NCT03928210(2019); NCT03889795(2019)

For the third strategy, the detailed molecular knowledge of MET regulation will provide opportunities to curtail this event and prevent the development of metastasis, which is of high clinical relevance. Depending on the clinical scenario, MET-inducing/stabilizing factors may inhibit metastasis if they block the initial EMT stages that allow the dissemination, or promote the later stages of metastasis, which can cause some conflicting considerations (van Denderen and Thompson, 2013; Pattabiraman and Weinberg, 2016). An emerging challenge is then to determine the correct timings for therapeutic interventions, and also to decipher correctly the contribution that intermediate states of the EMT spectrum make to tumor evolution for therapeutic interventions (McGranahan and Swanton, 2015). A high-throughput screening approach is required to identify suitable drugs or “repurposable” small molecular agents in context of specifically targeting hybrid and/or partial EMP cells. The concept of intermittent dosing (drug holidays) is also resurfacing to prevent the plasticity and transitioning of cells in carcinoma. For example, resistance to the BRAF inhibitor vemurafenib in melanoma is remodeled to forestall drug resistance (Das Thakur et al., 2013). Thus, the development of combinatorial therapeutic interventions that can target dynamics and plasticity alongside proliferative tendency of cancer cells may pave the way to more promising treatment strategies.

## Conclusion and Perspectives

The crucial roles of EMT-MET during embryogenesis and organogenesis is hijacked during tumor progression and metastasis. The roles of various signaling cascades, intrinsic and extrinsic mechanisms, and regulators that contributes to EMP dynamics are reasonably well determined, but more refined studies and techniques need to be employed to recapitulate the MET behavior of cells while extravasating, seeding and colonizing at secondary niches. The intricacies associated with phenotypic plasticity, stemness and intratumoral heterogeneity further sheds light on several unresolved queries. The reliable features of cellular behavior relating to drug persistent states through the spectrum of EMT need to be verified. Is there a ubiquitous molecular feature of partial/hybrid EMT cells that can be identified and targeted across all different cancer context types? What actual mechanisms do cancer cells employ to intravasate from the primary sites, and how do EMT - MET programs cooperate to assist cancer cells through several stages of cancer progression? We are still lagging in obtaining a wider and more complete understanding of the contributions of EMP in cancer. The sophisticated developments in lineage tracing using confetti animal models and implementation of other novel technologies such as high-resolution intravital imaging, live cell imaging, inducible reporter systems and single-cell sequencing techniques will provide great avenues in the fields of plasticity and dynamics around EMP. Finally, it is imperative to determine how phenotypic plasticity can be exploited, as therapeutic interventions that push the conversion of cancer cells to fat cells or apoptosis, for example, (David et al., 2016; Ishay-Ronen et al., 2019) might be promising approaches in clinical settings.

## Author Contributions

SB wrote the manuscript, developed the first draft, and contributed significantly toward development of the whole manuscript. PW contributed significantly in content material, figures and tables preparation and drafted metabolomics section. AT contributed in providing content material and drafted CTC section. ET refined, edited the manuscript, and approved the final version to be published.

## Conflict of Interest

The authors declare that the research was conducted in the absence of any commercial or financial relationships that could be construed as a potential conflict of interest.
